# Emerging Trends in Non-Protein Amino Acids as Potential Priming Agents: Implications for Stress Management Strategies and Unveiling Their Regulatory Functions

**DOI:** 10.3390/ijms25116203

**Published:** 2024-06-04

**Authors:** Kincső Decsi, Mostafa Ahmed, Roquia Rizk, Donia Abdul-Hamid, Gergő Péter Kovács, Zoltán Tóth

**Affiliations:** 1 Institute of Agronomy, Georgikon Campus, Hungarian University of Agriculture and Life Sciences, 8360 Keszthely, Hungary; roquiaibrahim@gmail.com (R.R.); toth.zoltan@uni-mate.hu (Z.T.); 2 Festetics Doctoral School, Institute of Agronomy, Georgikon Campus, Hungarian University of Agriculture and Life Sciences, 8360 Keszthely, Hungary; mostafa.ahmed.abdelmagid@agr.cu.edu.eg; 3 Department of Agricultural Biochemistry, Faculty of Agriculture, Cairo University, Giza 12613, Egypt; 4 Heavy Metals Department, Central Laboratory for The Analysis of Pesticides and Heavy Metals in Food (QCAP), Dokki, Cairo 12311, Egypt; donia.atalah11@gmail.com; 5 Institute of Agronomy, Szent István Campus, Hungarian University of Agriculture and Life Sciences, 2100 Gödöllő, Hungary; kovacs.gergo.peter@uni-mate.hu

**Keywords:** priming, non-protein amino acids, AABA, BABA, GABA, abiotic stress, biotic stress

## Abstract

Plants endure the repercussions of environmental stress. As the advancement of global climate change continues, it is increasingly crucial to protect against abiotic and biotic stress effects. Some naturally occurring plant compounds can be used effectively to protect the plants. By externally applying priming compounds, plants can be prompted to trigger their defensive mechanisms, resulting in improved immune system effectiveness. This review article examines the possibilities of utilizing exogenous alpha-, beta-, and gamma-aminobutyric acid (AABA, BABA, and GABA), which are non-protein amino acids (NPAAs) that are produced naturally in plants during instances of stress. The article additionally presents a concise overview of the studies’ discoveries on this topic, assesses the particular fields in which they might be implemented, and proposes new avenues for future investigation.

## 1. Introduction

Plants are regularly exposed to environmental stimuli, which they must respond to through cellular signaling pathways. In all cases, they must provide answers that effectively serve the adaptation mechanisms of the plant. When environmental stimuli exceed their tolerance threshold, plants may exhibit specific defense responses under exceptional circumstances. Stress can either trigger or induce inherent reactions in plants. The plant’s inducible responses allow it to efficiently respond to the effects of stress while conserving energy [1], but with high efficiency and adequate speed [2,3,4,5]. Living organisms consistently experience defense as a draining and energy-demanding process, which is why plants have evolved protective systems that only engage in challenging circumstances. Several defensive systems in plants function as a form of stress memory [6]. These energy-saving defense mechanisms are collectively known as plant priming.

Priming refers to the phenomenon where a plant, after being subjected to mild stimulating stress, becomes able to activate its defense mechanisms when faced with a more severe challenge. This may be seen as the plant developing a natural memory for stress [7]. The significance of the stress memory established in plants resides in the plant’s ability to conserve resources during periods without stress. Nevertheless, when a novel stressor emerges, the plant’s inducible reactions are promptly activated and require relatively low energy expenditure due to the stress memory induced by priming [6,8]. Defensive priming processes are equally suitable for mitigating damage caused by biotic [9,10,11,12,13,14,15] and abiotic [16,17,18,19,20,21,22] stress effects.

The plants can retain the memory of stressful experiences for an extended time. When needed, it can recall this memory, known as priming. The plant’s ability to retain long-term memory without a nervous system relies on the information and mechanisms encoded in the chromatin files [23,24,25]. However, plants can not only “remember” but also “forget”. They can store needed and delete unnecessary stress memory elements. The central element of all these processes is the autophagy. Proteasomes play a role in the establishment of stress memory as well as in “forgetting” processes. The proteins that are formed and/or modified as a result of the effects of stress store the information, that is, together they create the stress memory. Autophagy of proteins that are no longer needed causes the “forgetting mechanism” in the plant, while in some cases it can also activate “memories” [26]. Nevertheless, autophagy by itself is inadequate for the processes to take place; rather, the synchronized functioning of an intricate array of systems is required to trigger or suppress stress memory. Examples of such systems include plant hormones, reactive oxygen species (ROS), non-protein amino acids (NPAAs), etc. [27].

The Impact of amino acids and their derivatives on plant responses to both biotic and abiotic stress is discussed in a comprehensive review by Cai and Aharoni (2022) [28]. Plant organisms contain alpha-, beta-, and gamma-aminobutyric acids, among other non-protein amino acids. Their endogenous presence and quantitative increase can be linked to abiotic and biotic stress effects.

The endogenous role of AABA is little known; however, numerous studies have revealed the importance of the natural presence of its two isomers (BABA and GABA) in the plant organism. BABA present in endogenous form is found in moss [29] and in some plant species, e.g., in Arabidopsis [30] and maize (Shaw et al., 2016) [31]. Thevenet and his colleagues (2017) noticed that BABA is present in a low amount but in endogenous form continuously in Arabidopsis leaves [32]. They also recognized that this is the least abundant endogenous amino acid of the three NPAAs, while AABA is around 60 times and GABA is 1200 times BABA. It is present in higher amounts in the leaves than in other tissues, e.g., in the root [32].

In 2014, Luna et al. discovered the IBI1 receptor protein, the presence of which confirms the assumption that different abiotic or biotic stressors can induce an increase in the endogenous level of BABA in the presence of the protein, thereby playing a role in the activation of defense responses. There is evidence that the level of endogenously present BABA increases rapidly through ABA-dependent signaling mechanisms during drought and salt stress [33], as well as during attacks by various pathogens [34]. Balmer et al. (2019) investigated the quantitative increase in endogenous BABA in tomatoes after infection and found that the increase in the level of the endogenously present amino acid is primarily local and mainly concentrated at the site of infection, while exogenously administered BABA moves relatively easily and quickly in the plant organism [35]. They also discovered that the level of BABA is different in each tissue, typically the lowest in the roots, medium in the leaves, and the highest in the reproductive organs.

GABA, which is produced endogenously in the plant organism, is an important intermediate product of nitrogen metabolism and thereby indirectly affects amino acid biosynthesis [36]. It participates in the construction of carbon skeletons [37] and provides energy for biosynthetic processes. GABA is also involved in signaling or regulatory mechanisms [38]. It is involved in plant growth and development processes [39,40,41]. The biochemical process of action of GABA has been elucidated in detail [42].

However, it is also known that, for decades, research has focused on the exogenous application of these bioactive compounds; moreover, the examination of their priming effects has been the focus of research for decades. Non-protein amino acids (AABA, BABA, and GABA) work excellently as priming compounds; however, at the same time, they do not activate all the defense mechanisms before the onset of stress but only prepare the plants in an energy-saving manner to ward off possible strong damaging effects [43].

Plant immunity can be triggered even in the absence of significant abiotic or biotic stress factors. The early endeavors originated from the finding that stress-induced impacts on plants elicit cellular responses. These can serve as the initial catalyst for the synthesis of specific molecules of defense within plant cells. Protective compounds include phytohormones such as salicylic acid (SA), jasmonates (JA), abscisic acid (ABA), ethylene (ET), brassinosteroids, as well as vitamins, oligogalacturonides, volatile organic compounds, azelaic and pipecolic acid, reactive oxygen, nitrogen, and sulfur species (RONSS), acetic acid, ethanol, and non-protein amino acids such as alpha-, beta-, and gamma-aminobutyric acid [44,45,46,47,48]. As a result of both biotic and abiotic stress effects, certain biochemical pathways are strongly induced (e.g., SA, JA, ET pathways, etc.), which encourage plants to produce bioactive metabolites [49,50,51,52,53,54,55,56,57]. These compounds are involved in the activation of cellular defense mechanisms.

**Figure 1:**
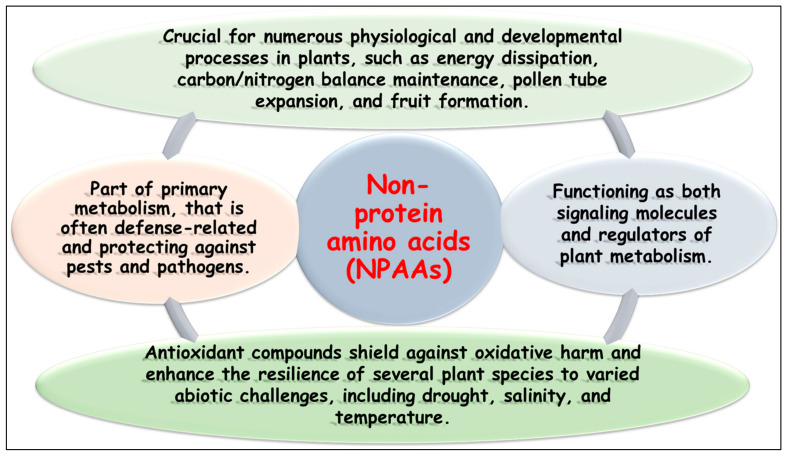
The different regulatory effects of the non-protein amino acids (NPAAs) in plants.

The examination of various protective natural compounds made by plants in response to stress led to the testing of the external, synthetic application possibilities of these compounds [9,58,59,60,61,62]. The effectiveness of the exogenous application of these compounds has already been confirmed by several reports [63,64,65,66]. The present review considered recent studies that investigated the impacts of externally administered non-protein amino acids (AABA, BABA, and GABA). Researchers have utilized these acids as initiators to counteract the negative impacts of abiotic and biotic stress. By doing so, we can help in structuring the explanation of the priming mechanisms and assessing the efficacy of individual NPAAs during their evaluation (Figure 1).

## 2. Discussion

### 2.1. Plant Defense Mechanisms and Priming Reaction

Plants exhibit the distinctive feature of individual plant cells’ capability of autonomously defending themselves and executing both general and particular defensive mechanisms. A dual-layer model can characterize the plant’s immunity and defense capacity. Each plant possesses an inherent and constant primary defensive mechanism against various stressors. Natural barriers, such as the cuticle layer and hairiness, can serve as lines of defense against stress by effectively preventing its harmful effects. This layer of protection is commonly known as general, natural, or innate immunity. The second line of defense, called the specific immune response reaction, is triggered by various environmental factors. The term “specific, adaptive, or acquired immunity” is used to describe the latter [67]. Pathogen attacks can link to the discovery of active, inducible plant responses. The plant recognizes the pathogen’s triggering elicitor, which forms the basis of its formation mechanisms. Elicitors are molecules produced or derived from the surface of microbes that can trigger a plant response [68]. Other stimuli, in addition to microbial attacks, can trigger a series of possible complex and interrelated stress responses that make up an inducible defense [69].

Only when a plant detects an emerging danger does it activate and form its secondary line of defense. A change in the intensity of many biochemical processes indicates the activation of the secondary line of defense. Initiating biochemical processes at the cellular level triggers various responses that aid plant defense. Such responses include, for example, the activation of the antioxidant enzyme system, the production of various antimicrobial compounds, the strengthening of the cell wall, and so on. Plants normally induce their secondary defense lines, pattern-triggered immunity (PTI) and effector-triggered immunity (ETI), in response to stress effects such as fungal pathogen attacks. PTI, the first mechanism of defense, usually results from the recognition of microbes, and ETI represents the second, but even stronger, layer of defense [70].

Mobile defense signals systematically transmit the induced immunity in the plant from the local tissue (the infected part) to the not-yet-infected tissues, providing long-term protection against a wide spectrum of pathogens. This immunity affecting the entire plant organism is called systemic acquired immunity (SAR) [71]. Non-pathogenic beneficial microbes can induce Induced Systemic Resistance (ISR), a form of systemic immunity [72]. Rather than directly inducing the defense machinery, ISR-conditioned plants may induce faster and/or stronger defenses during subsequent pathogen interactions. This sensitization mechanism is called priming [73].

Numerous artificial chemicals can trigger the priming reaction. Cui et al. (2014) successfully applied artificial SA derivatives, without claiming to be comprehensive, against various fungal pathogens in cucumbers [74]. Isonicotinic acid (INA) and its derivatives [75,76,77,78], benzothiadiazole (BTH) [79,80,81], and synthetic jasmonic acid (JA) [82,83,84] also belong to priming chemicals. In addition to the above compounds, benzoyl salicylic acid (BzSA) has been successfully used, among others [85], as well as beta-aminobutyric acid (BABA) [86].

### 2.2. Priming with Alpha Aminobutyric Acid (AABA)

**Figure 2:**
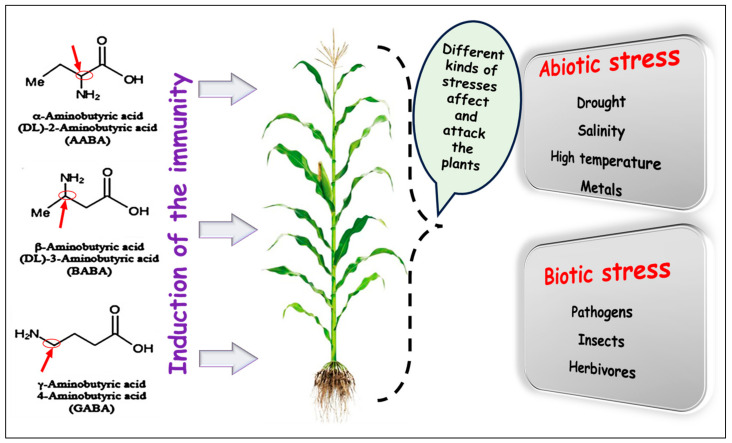
Representation of the chemical structures of α-, β- and γ-aminobutyric acid enantiomers, standing against the harmful effects of abiotic/biotic stress in plants. The red arrows indicate the carbon atoms attached to the amino groups, forming the different isomers of butyric acid. The image of the maize crop is just an example.

All three of the best-known representatives of NPAAs are organic carboxylic acid derivatives. Among the compounds containing four carbon atoms, AABA contains an amino group on the second, BABA on the third, and GABA on the fourth carbon atom [87]. Figure 2 shows the three versions that can be considered isomers of each other, facing the harmful effects of abiotic and biotic stress [40,88]. More than one study on sunflower [89], pepper [90], grapes [91], cauliflower [92], and lettuce [93] has investigated the effectiveness of BABA, and they all came to the same conclusion: only the presence of the amino group in position 3 makes butyric acid active, i.e., they made it clear that the other isomers are ineffective in priming. Based on the research, it appears that the amino group’s position determines the compounds’ biological activity and usable priming ability [92,94].

Over the years, the studies that revealed the use of NPAAs as external priming compounds increased. At first, it was believed that while BABA might be effective in inducing resistance mechanisms against biotic stressors, its two known isomers, AABA and GABA, were ineffective [92,94]. Over the years, many conflicting results have been recorded regarding the priming effect of butyric acid isomers. Silué et al. (2002) found that priming cauliflower plants with AABA did not produce results against the pathogen *Peronospora parasitica* [92]. After that, Cohen et al. (2010) obtained the test result that neither AABA nor GABA is effective against the pathogen *Bremia lactucae* [93]. The authors justified this fact by saying that the location of the amino groups in the isomers is decisive in terms of activity [86]. In their study, Lotan and Fluhr (1990) discovered that the application of DL-α-aminobutyric acid (AABA) to entire tobacco leaves produced a similar outcome as tobacco mosaic virus (TMV) in terms of activating PR proteins. They utilized the AABA technique on tobacco plants, expecting the resulting PR proteins to protect against downy mildew caused by *Peronospore tabacina*. As this was not the situation, they attempted the other two isomers of aminobutyric acid, namely, DL-β-aminobutyric acid (BABA) and γ-aminobutyric acid (GABA) [95].

Siegrist et al. (2000) observed that BABA has an excellent priming effect against TMV in tobacco plants [96], AABA has less of an effect, but GABA proved to be completely ineffective. Gur et al. (2021) conducted a preventive priming inoculation against the pathogen *Alternaria alternata* in apples and found that AABA was ineffective against the pathogen-caused rot [97]. In their study of the flowering plant *Liliodendron chinense × tulipifera* in 2021, Wang et al. (2021) found that the priming compound AABA did not alter gene expression. This was unexpected, but it did not affect the level of citrates, which is an important defense against aluminum stress [98].

Nevertheless, multiple investigations have refuted that AABA is ineffective as a priming chemical. While the extent may vary, all three isomers have demonstrated an active preparative impact [99,100]. Šašek et al. (2012) compared *Brassica napus* plants primed with the three isomers in terms of *Leptosphaeria maculans* symptoms. They found that the size of the lesions caused by the pathogen was significantly reduced by 55%, even when AABA was applied [101].

Grochala and Kępczyńska (2013) found that among the three isomers, BABA was the most effective (77%), but priming application of AABA (55%) and GABA (20%) also reduced the symptoms of *Leptosphaeria maculans* in grapes [87]. Fu et al. (2017) found that AABA did not have a direct effect on the fungal infection caused by *Penicillium expansum*. While the visible signs of infection directly decreased following the priming with the other two isomers, the effect of AABA can be said to be more indirect, but it can be verified since an increase in the activity of the peroxidase enzyme was observed following its application [102]. In a different study, Kim et al. (2019) examined how well the three priming isomers protected pine seedlings from exposure to *Bursaphelenchus xylophilus* bacteria. They observed that priming with all three isomers of butyric acid led to higher gene expression levels in some members of certain peptide families. This resulted in the activation of chitinase, glucanase, and ribonuclease activity, as well as an increase in the level of some antimicrobial peptides [103]. In general, the study on AABA is not as extensive as that on other isomers of butyric acid. This is mainly due to the popular belief that AABA is ineffective for priming. Nevertheless, the situation is more complex than initially thought. The current research suggests that the specific plant species, the type of stress it targets, and the dosage influence the alpha-isomer’s impact. Table 1 provides some plant species that have undergone research to comprehend the role of ABAA in stress tolerance and regulation.

### 2.3. Priming with Beta Aminobutyric Acid (BABA)

Because of its well-known priming effectiveness, research on beta-aminobutyric acid has become widespread. Table 2 lists several plant species that have undergone investigation to better understand the role of BBAA in stress tolerance and regulation. Pastor et al. (2014) and Slaughter et al. (2012) studied the idea that using BABA for priming leads to higher gene expression, mostly in the production of PR proteins and primary and secondary metabolites [104,105]. It was hypothesized that the enhanced defense response triggered by BABA could be passed down from one generation to the next in plants. Baccelli and Mauch-Mani (2016) demonstrated that the beta isomer of aminobutyric acid activates numerous metabolic pathways. Consequently, it can assist plants in coping with both biotic and abiotic stressors by functioning as an external bioactive priming molecule. BABA has been demonstrated to stimulate signaling pathways such as inositol (PtdIns), phenylpropanoid (PP), salicylic acid-dependent pathways (SA), abscisic acid (ABA), and ethylene (ET) [34]. Wilkinson et al. (2018) studied soil injection and plant conditioning with BABA to evaluate the priming effects of this isomer involving tomato plants. The injection of soil positively changed the microbiome; metabolomic analyses showed that defense-boosting microbes became more common and stayed there for a long time in the treated soil. In addition, primed plants were less susceptible to *Botrytis cinerea* infection [106].

Wang et al. (2019) also studied the pathogen *Botrytis cinerea* and discovered that priming treatment with BABA stimulated SA-dependent signaling mechanisms. They also activated the key enzymes of the pentose phosphate pathway (PPP) and the ascorbate glutathione cycle (AGC) [108]. This change led to the strengthening of cellular detoxification processes, the increased presence of reduced glutathione (GSH), reduced nicotinamide adenine dinucleotide phosphate (NADPH), and an increase in the proportion of PR proteins. Mango fruits were also treated with BABA, and it was found that the priming had a strong beneficial effect on the destructive effects of the pathogen *Colletotrichum gloeosporioides* [109]. The pretreatment induced a positive response in the defense-related signaling pathways at the transcriptional and proteome levels, as well as in the formation of secondary metabolic products.

Coss-Navarrete et al. (2020) investigated the priming effect of BABA in beans and established that the externally applied compound can induce systemic acquired resistance (SAR) in the presence of biotic stress factors [122]. Li et al. (2022) concluded that priming with BABA beneficially affected peach defense mechanisms against the pathogen *Rhizopus stolonifer* by enhancing ABA production, callose formation, and oxidative burst [110]. A similar conclusion was previously proved by Zimmerli et al. (2000) in BABA priming experiments with the *Arabidopsis thaliana* model plant [111].

Additionally, the research broadened its scope to encompass a wider range of topics, leading to the external application of butyric acid derivatives to combat not only living organisms but also non-living stressors. Karimi et al. (2017) primed pistachio plants against salt stress using BABA, and they concluded that the proline level of the leaves and the stability of the cellular membranes increased as a result of the treatments [112]. Mohamadi et al. (2017) investigated the behavior of rapeseed plants during drought stress, after priming with BABA [113]. The study revealed an increase in the proportion of reduced glutathione (GSH), which facilitated the initiation of cell detoxification processes. Moreover, the amount of lipid peroxidation decreased in the plants that were given BABA. This meant there was less malondialdehyde (MDA) in the cells of the treated plants compared to the drought-stressed plants that were not given BABA. Simultaneously, the cells experienced a decrease in hydrogen peroxide (H_2_O_2_) levels, indicating that the BABA pretreatment not only alleviated drought stress but also significantly prevented secondary oxidative stress [113].

Shehu and his colleagues compiled a summary of the enhancing effects of BABA priming that were already known. They also found that rapid defense gene expression enhances the efficiency of photosynthesis, improves the stability of cell membranes, and helps maintain a balanced water level [123]. Ma et al. (2020) treated tobacco seedlings with BABA to prevent chilling stress. The expression level of some genes closely related to chilling stress increased, confirming its creation and effect. The results of the BABA priming treatment showed that the chlorophyll content of the plants increased and that they were generally better able to withstand the harmful effects of low temperatures [114]. It was described as a novelty that BABA, combined with a dosage of Ca^2+^, seemed to extend the priming effect. The combined administration of the two components increased the activity of antioxidant enzymes, thereby reducing the harmful effects of secondary oxidative stress, as well as increasing the abscisic acid and auxin content of the plants.

Another study by Mahmud et al. (2020) found that priming with BABA protects against salt stress in two ways. First, it stops plants from taking in too much Na+. Second, it improves the activity of the antioxidant system, specifically, ascorbate-peroxidase (APX), catalase (CAT), dehydroascorbate reductase (DHAR), glutathione reductase (GR), glutathione peroxidase (GPX), glyoxalase I and II (GLO I and II), monodehydroascorbate reductase (MDHAR), and SOD enzymes [115]. Chickpea seeds were also treated with BABA to protect them from salt stress. Early treatment helped the seeds germinate because they were able to take in water normally even though they were in a salty environment. As a result of the treatment, the quantitative parameters (root and shoot length) of the productive plants also developed favorably [116].

Under heat stress, Quan et al. (2022) investigated the priming effect of BABA in Chinese cabbage [117]. BABA significantly increased the cells’ antioxidant capacity and increased the levels of APX, CAT, peroxidase (POD), and superoxide dismutase (SOD). Additionally, the membranes’ characteristic degree of electrolyte leakage during stress has decreased, indicating no damage to the membranes. Since the membranes remained largely intact, there was no harmful increase in MDA levels. According to their findings, priming’s beneficial effect extended to the determining parameters of photosynthesis, such as chlorophyll content and chlorophyll fluorescence [117].

Following the use of BABA as a priming treatment for Linum usitatissimum, it was observed that the plants became more resistant to drought stress. Priming primarily led to a significant increase in antioxidant capacity, as evidenced by the increased levels of APX, CAT, SOD, and POD enzymes [118]. Bhutta et al. (2023) suggested that the most appropriate concentrations of BABA for priming are 2–3 mM in drought stress. These concentrations were already effective enough to boost levels of antioxidant enzyme production under stress [119]. Abdulbaki et al. (2024) treated pepper seeds against drought stress with externally administered BABA and then examined any potential beneficial effects [120]. The changes at the cellular level showed that the BABA treatment had an excellent effect on the plants, even in the case of early seed treatment. Proline content increased in mature plants, which is known to be an effective osmolyte for alleviating drought stress. In addition, the level of antioxidants increased, resulting in a decrease in ROS accumulation, which prevented lipid peroxidation and the accumulation of MDA derived from it. Furthermore, BABA treatment prevented chlorophyll degradation under drought stress. Plant physiology also underwent positive changes; despite stress, the plant’s height and leaf surface increased, and its water balance stabilized [119].

There are no relevant references available on the subject of AABA; however, several research groups have already published on the role of BABA in stress memory [6]. In BABA-primed plants, PR genes were also activated upon repeated crossing of a lower stress threshold, even months after the priming treatment [33]. Hulten et al. (2006) showed in *Arabidopsis* that after pretreatment of the mother plant with BABA, seed germination, and emergence were slower; however, their resistance to some phytopathogenic pathogens increased [107]. In addition to the positive effects, it must be mentioned that—although there is no information on this topic about AABA—according to studies, the use of BABA, which is more often used for priming, is highly dependent on plant species and dosage [43]. At higher doses (1–10 mM), BABA can cause toxic symptoms in the leaves, while, when applied to the soil, it can inhibit root growth [33,43,121]. In some experiments, treatment with too high or repeated doses of BABA resulted in the sterility of female flowers [124]. Since BABA is also an amino acid, excessive exogenous use can inhibit protein biosynthesis, thereby negatively influencing cell growth and adversely affecting the functioning of generative organs. Some researchers [125,126,127,128] attribute these effects of BABA to intervention in the plant amino acid transport system.

Several observations have also been made that necrotic lesions [100] or even complete leaf death [92] occurred with the application of BABA. The probable reason for this was the phytotoxicity resulting from the high concentration, which could cause metabolic disorders [107]. Applying it to the root reduced the mass of the root, the shoot length, and the root diameter. The authors explained all of this by the fact that the additional energy requirement (ATP) needed to activate protective compounds takes resources away from growth and development processes [129].

### 2.4. Priming with Gamma-Aminobutyric Acid (GABA)

As research progressed, the focus also shifted towards GABA, which is the third isomer of non-protein amino acids. Aside from AABA, this was the alternative compound initially perceived as ineffectual or minimally useful. Today, we have debunked this idea and, based on referenced data, found that this is the most extensively studied amino acid among the three isomers. Studies on the benefits of naturally accumulating GABA in plants began at the beginning of the decade. Table 3 displays many plant species that have been studied over the years to figure out the role of GABA in tolerating or regulating the variant types of stresses. The GABA shunt primarily synthesizes GABA, but the polyamine biosynthesis pathway can also form it. The citric acid cycle forms glucose, which -ketoglutarate metabolizes and -oxoglutarate transaminase (GABA-T) transaminates into glutamate. Glutamate decarboxylase (GAD) then decarboxylates glutamate to form GABA [130].

Kinnersley and Turano (2000) discovered that plants initiate GABA accumulation through two distinct mechanisms in response to stress-induced effects [159]. Firstly, rapidly changing abiotic stress effects (temperature, wind, etc.) cause a sudden increase in the Ca^2+^ level, which, forming a complex with calmodulin, will indirectly affect the increase in the GABA level. Secondary messengers transmit the stimulus from the external environment inside the plant cell. Second messengers aim to trigger intracellular signaling pathways, amplifying the signal and triggering a cellular response through the activation or inhibition of transcription factors. Receptor activation releases second messengers (including Ca^2+^) into the cytosol, which, in turn, affect numerous intracellular enzymes, ion channels, and transporters. The increase in the Ca^2+^ level in the presence of calmodulin affects vital processes. Calmodulin can bind two calcium ions at the end of its chain [160], thereby creating an active complex. In its active state, the complex can bind to calcium-dependent protein kinases (CDPKs). These proteins then activate the function of additional enzymes and promote the expression of individual genes by phosphorylating transcription factors [159]. Other types of abiotic stressors (heat, cold, salinity, or drought) initiate the endogenous synthesis of GABA via a different pathway. These stressors directly cause a strong pH drop in the cytoplasm, which indirectly increases the level of GABA in the cell. The activation of the enzyme glutamate-decarboxylase (GAD) in both pathways ultimately initiates GABA synthesis [156].

According to Yuan et al. (2024), plants develop stress memory as a result of repeated stress effects, including the activation of the GABA system [161]. Since an increase in endogenous GABA levels can trigger defense in plants, its exogenous application is also becoming more widespread. Vijayakumari and Puthur (2016) exposed black pepper (*Piper nigrum*) plants to osmotic stress using polyethylene glycol (PEG) treatment [43]. GABA priming also increased the amount of endogenous GABA that plants produced. After priming, lipid peroxidation was demonstrably reduced, and photosynthesis and cellular respiration activity were less inhibited. Wang et al. (2017) studied the effect of exposing corn plants to moderate and strong salt stress, which mainly caused significant damage to their life processes [134]. The authors experimented with the application of exogenous GABA as priming to mitigate all the damages that appeared. The exogenous GABA influence increased the activity of the GAD enzyme and increased the production of endogenous GABA in the plants, indicating a perfect substitution of the two compounds, proving the effectiveness of the exogenous application. This fact is also supported by the fact that the primed plants tolerated the attack of the stressor better in all respects than their untreated counterparts [162].

As GABA levels rose, the ability of antioxidant enzymes to do their function increased, and the membranes remained intact. This means that the harmful effects of oxidative stress decreased. An example of this is the decrease in the level of MDA, which is a metabolite that is a byproduct of membrane lipid peroxidation caused by oxidative stress. Additionally, the salt load reduced the drought stress by increasing the proline level, an osmolyte. GABA also had a positive effect on photosynthetic processes. Wu et al. (2020) obtained similar results when they exposed tomato plants to salt stress [135]. Exogenous GABA treatment increased the expression of *Solanum lycopersicum* GAD (SlGAD) genes increased as a result of exogenous GABA treatment. Thus, the activity of the GAD enzyme increased despite stress. Furthermore, the level of certain amino acids (for example, proline) increased. As a result of the combined effect, GABA-primed plants showed stronger growth, more efficient photosynthesis, and greater green mass growth, even in the case of salt stress. On the other hand, GABA priming had a beneficial effect on the nitrogen metabolism of creeping bentgrass plants under water-stress conditions [136]. The authors in the previous study revealed that GABA promotes the functioning of nitrite reductase (NIR) and glutamine synthetase (GS), thereby positively influencing nitrogen metabolism. In addition, priming increased the activity of the enzyme glutamate dehydrogenase (GDH), which promoted the functioning of the citric acid cycle and glutamate transformation [136].

Rezaei-Chiyaneh et al. (2018) investigated the effect of GABA priming in black cumin plants under a water deficit [137]. In addition to the significant increase in chlorophyll levels, the levels of the osmolyte proline and CAT enzyme also increased, which, overall, had a beneficial stress-relieving effect on the plants. Cheng et al. (2018) investigated the possibilities of external application of GABA in the white clover to alleviate the harmful effects of salt stress [138]. The priming treatment resulted in an acceleration of starch breakdown in plant seeds under salt stress, attributed to the enhanced activity of the amylase enzyme. Barley analysis also revealed a stimulated amylase activity in the aleurone layer [139]. Additionally, the previous study proved that the expression of many genes encoding Na^+^/K^+^ transport, antioxidant enzymes (APX, CAT, Cu/ZnSOD, FeSOD, MnSOD, GPX, glutathione-S-transferase (GST), and MDHAR), and some dehydrin (DHN) genes, was improved under the influence of GABA [139]. DHNs are considered a potential genetic underpinning of drought tolerance. Thus, the authors found new evidence of the protective effect mediated by GABA. Combining these effects, the viability, osmotic stress tolerance, and growth vigor of germinating seeds and seedlings improved. Similar research was conducted with similar results in mungbean and fresh-cut pumpkins during storage [140,142]. The post-harvest state of pumpkin seeds was also examined and it was established that GABA priming also effectively prevented any unsaturated fatty acid (FA) degradation that may occur during storage [141].

Seifikalhor et al. (2019) proved that there is a positive effect of GABA on the physiological attributes of plants in case of salinity, hypoxia/anoxia, drought, temperature, heavy metals, plant–insect interactions, plant–microbe interactions, and ROS-related reactions [163]. Kaspal et al. (2021) summarized the role of GABA during abiotic stress conditions [164]. Dabravolski and Isayenkov (2023) summarized the results experienced with exogenous GABA priming under the effects of salt stress [165]. Hayat et al. (2023) discussed the importance of GABA in plant physiology and its application possibilities, primarily in horticultural crops [166].

Zhou et al. (2021) obtained data similar to the above results. The authors showed that even under water stress, GABA priming increased the expression of some dehydrin genes, as well as the expression level of genes encoding DHN content [143]. They also discovered, as a novelty, that GABA priming induces some transcription factors (dehydration-responsive element binding—DREB family) in white clover under drought stress. It was proved that the DREB gene family is one of the most important transcriptional regulators that help plants tolerate abiotic and biotic stresses [144]. There was also an increase in the level of antioxidative enzymes and scavenging functions, which were identified in the primed plants. The previously described antioxidant genes (CAT, SOD, APX, POD, DHAR, MDHAR, GR) that respond positively to GABA priming also showed a similar increase. Similar results were obtained in the case of exposure of creeping bentgrass to heat stress [145], water stress [146], water stress in maize [147], and chilling stress in tomato [148].

Li et al. (2018) achieved additional favorable outcomes in creeping bentgrass plants when subjected to both heat and drought stress using GABA priming [149]. Many genes and transcription factors, including mitogen-activated protein kinase (MAPK, which participates in signal transduction), WRKYs (which are transcriptional factors that bind DNA), MYBs (which are proteins that include the conserved MYB DNA-binding domain), heat shock proteins (HSPs, ubiquitous proteins that can save cells under multiple complex stress conditions) [167], DHN, abscisic acid-responsive transcription factors (ABFs, which are ABA-signaling components that participate in abiotic stress response), and mitochondrial genes (MTs), as well as gene expressions encoding antioxidant enzymes, were increased by the priming treatment [149]. The investigations of the combined effects of drought, heat, and salt stress in creeping bentgrass were extended [168]. Polyamines (PAs) are produced in nature at all levels of the organs of living cells. They significantly increased in GABA-primed plants; however, each stressor activated different PAs. Accumulation of putrescine was observed in the case of drought, arginine during salt stress, and spermine during heat stress. It is known and proved that the increase in polyamine levels in plants is one of the most typical and most common responses to stress [45]. The polyamine concentration also increases due to a lack of water, lack of nutrients, acidification, and high salt concentration [169]. Takahashi and Kakehi (2010) reported that polyamines influence membrane stability and permeability by interacting with the membrane functional groups. By binding to DNA and RNA, they stabilize their secondary structure and protect these molecules against enzymatic degradation. The fact that cell division halts in their absence demonstrates their importance, as DNA cannot replicate without them. An increase in their quantity positively correlates with an increase in the intensity of protein and nucleic acid synthesis during cell division [170].

Li et al. (2020) also found that GABA priming increased the levels of some amino acids under combined stressors. Treatment with exogenous GABA activated the GABA shunt, and the levels of amino acids (glutamic acid, alanine) and GABA increased. Levels of aspartic acid, glycine, and phenylalanine also increased. This fact suggests that abiotic stress effects are commonly regulated [168]. Additionally, it is important to note that each of the three stressors can cause individual changes. Firstly, in the case of drought and heat stress, the level of methionine increased, while the levels of cysteine, threonine, and serine changed in response to drought and salt stresses. However, examining only drought stress revealed an increase in proline levels following GABA priming [168].

Some other important changes have also occurred in carbohydrate metabolism as a result of priming. During drought stress, the levels of mannose increased; however, under high temperatures, fructans and sucrose levels were higher. Under salt-stress conditions, trehalose and xylose increased in GABA-based plants. The change in carbohydrate content caused by stress is particularly important in the life of plants since carbohydrate metabolism is related to photosynthesis, transport processes, and cellular respiration [171]. Among the water-soluble carbohydrates, sucrose and fructans play an important role in adaptation to stress. Sucrose can replace water, thereby helping cell membrane phospholipids to remain in the liquid crystalline phase [172]. Fructans are important reserve carbohydrates, more than the amount of starch in the stem and leaf. During osmoregulation, they play an important role in physiological processes induced by stress, particularly drought tolerance [173]. Mannose has some protective effects related to osmoregulation [174], and xylose was also characterized as an osmosensitive regulator [175]. There is also a promising application of trehalose for its exceptional bioprotective properties shown against drought stress and osmotic stress [176]. Similar results were found in creeping bentgrass [150] and in sunflower plants [151] when they were primed against a combination of heat and drought stress.

In addition to the data consistent with the above results, it was also described by Weber et al. (2008) that the expression level of certain HSPs, osmotin (a multifunctional stress-responsive, defense-related protein family, which is involved in inducing osmo-tolerance in plants) [177], and aquaporin (AQP; water and water/glycerol channels that are responsible for facilitating the rapid passive transport of water, solutes, or ions across biological membranes) [178] genes increased under the influence of GABA [179]. In plant cells, HSPs assemble into heat shock granules (HSGs) under high-temperature stress. HSPs and mRNAs compose these granules. Messenger RNAs (mRNAs) temporarily hide in these HSGs when stress effects occur, thereby avoiding stress effects such as denaturation due to high temperatures. At the end of the stress effect, the structure of HSGs loosens and connects with polysomes active in protein synthesis. The translation of mRNAs begins, and protein production increases, promoting rapid plant cell regeneration [179]. Ubiquitins are considered the cleaners of the cells, create conjugates (junctions) with aged enzymes that have lost their function, and detoxify the cytoplasm. They can be produced from proteins, peptides, or amino acids, which can be reused. The ubiquitin–protein complexes are then degraded by enzymes [180].

Bhardwaj et al. (2021) obtained some results from lentils under heat stress that were the same as those previously obtained by other research groups in other plants and under other abiotic stressors [152]. In addition to the already-known beneficial effects, the authors showed that the efficiency of pyrroline-5-carboxylate synthase (P5CS) and betaine-aldehyde dehydrogenase (BADH) enzymes increased as a result of GABA priming, which ultimately induced proline and glycine-betaine (GB) biosynthesis [152]. In addition to proline, glycine betaine is also an excellent osmoprotective compound. These facts are also confirmed by Abd El-Gawad et al. (2021), who primed bean plants with GABA against drought stress [153].

Further research confirmed that the effectiveness of GABA priming also extends to the components of photosynthesis, namely, to help the development of pepper plants (which were primed with GABA) grown under low light intensity. Priming led to a reduction in stress-induced depressions in net photosynthetic rate, stomatal conductance, maximum quantum yield of photosystem II (PSII) efficiency, electron transport rates, and photochemical quenching coefficient values [154]. *Vicia faba* plants were primed with GABA and found that the efficiency of photosynthesis in the primed plants improved even during osmotic stress using polyethylene glycol (PEG), sulfur dioxide stress, and salt stress. This could be due—among other things—to an increase in the leaf area, the size and number of stomata on the leaves, and the gaps between the stomatal openings [155]. Priya et al. (2019) examined the suitability of GABA as a priming thermo-protectant chemical in mung beans. The use of GABA priming has been shown to effectively enhance the fertility markers of plants, including pollen amount, pollen viability, pistil maturity, pistil fertility, and responsiveness, under conditions of heat stress [156].

Li et al. (2022) primed creeping bentgrass plants with GABA against heat stress and observed that the uptake and utilization of nutrients by the plants changed in a positive direction as a result of the priming compound [110]. The accumulation of some macro- and micro-elements had a beneficial effect on some indicators of plant photosynthesis and water balance characteristics, even under stress. This fact highlights that the priming compound itself does not always have a direct effect but that, sometimes, during stress, it can change the behavior of primary and secondary metabolites, thereby promoting the transfer of plant metabolic processes to alternative pathways and, ultimately, the activation of defense mechanisms [110]. Mahmud et al. (2017) examined the chromium-stressed indian mustard *(Brassica juncea)* plants that were primed with GABA. They observed that both enzymatic and non-enzymatic antioxidant capacity increased and further chromium uptake was inhibited in the plants [181].

Similarly, creeping bentgrass plants were investigated by Zhou et al. (2023), also during abiotic stress (heavy metal stress; aluminum) [157]. Plants primed with GABA against aluminum toxicity had more stable water balance and photosynthetic parameters than their untreated counterparts. Following the treatments, an increase in antioxidant capacity and the accumulation of some metabolites linked to responses to acidic aluminum stress were observed, including shikimic acid, some amino acids, mono- and disaccharides, and glucose-6-phosphate. The expression levels of genes related to malic acid and citric acid translocation increased.

When the plant tissues are damaged, infected, or under stress, they redirect the usual metabolic routes to alternate pathways. One instance of this is the cellular respiration process, where the focus shifts to the pentose phosphate pathway rather than glycolysis under stressful conditions. This process is considered direct oxidation, in which ribulose-5-phosphate is rapidly and abundantly formed from the accumulating glucose-6-phosphate. This molecule is directly linked to the Calvin cycle, which is necessary for photosynthesis’s dark reactions. Additionally, it is the initial compound required for the production of nucleic acid, which is essential for regeneration processes. Furthermore, vigorous operation of the pentose phosphate route, which diverges from it, can enhance the shikimic acid pathway. The shikimic acid pathway generates antibacterial chemicals that contribute to defense and regeneration. Therefore, these mechanisms generate the necessary intermediates for defense and regeneration, as exemplified by the aforementioned metabolites. Some research groups are also looking into the possibility of using GABA as a priming agent to make the body more resistant to infections, in addition to testing it against abiotic stressors. Studies have demonstrated that pathogens can cause the buildup of naturally occurring GABA in plants [108,182,183,184,185,186]. Simultaneously, researchers initiated parallel investigations into the potential application of exogenous GABA to combat biotic stresses. The growing research on the impact of GABA has yielded increasingly abundant findings on its efficacy in combating biotic stresses [131,132,187].

In their comprehensive study, Tarkowski et al. (2020) investigated the mechanisms associated with biotic stressors in the external administration of GABA. Research has demonstrated that GABA can serve as a potent priming component, aiding the defense against infections by mitigating the subsequent oxidative stress that arises after an attack. Furthermore, it fulfills a crucial intermediary function in nitrogen and carbon metabolism, as well as in creating the connection between them via the GABA shunt [187]. In 2020, Janse van Rensburg and Van den Ende looked into GABA as a stress-inducible non-proteinogenic amino acid. They discovered that it can kill Botrytis cinerea even at low concentrations (up to 100 µmol) because it starts antioxidant processes and especially boosts the activities of catalase and guaiacol peroxidase enzymes. The plants primed this way showed low H_2_O_2_ concentrations, even in the presence of infection. Furthermore, we assume that GABA participates in the carbon and nitrogen cycles as one of the molecules involved in signaling mechanisms [133].

Finally, Cheng et al. (2023) performed a comprehensive transcriptomic analysis in apples following GABA priming. A total of 1271 genes were identified in apples that were demonstrably differentially expressed following GABA priming during drought stress compared to the untreated control [158]. Most of these genes contribute to carbon metabolism, the MAPK signaling pathway, glutathione biosynthesis, and the formation of secondary metabolites. This supports the fact that GABA priming increases the activation of defense mechanisms in plants. Overall, it can be concluded that using GABA as a priming compound is still more widespread against abiotic stressors.

The role of GABA in stress memory has been proven in drought stress mainly in connection with the regulation of stomatal closure [188] and the increase in endogenous GABA production [161]. Limitations of the exogenous use of GABA were only shown when very high concentrations were administered, but this was also species-dependent. While in *Stellaria* species growth was already inhibited above a concentration of 1 mM [189], a concentration of 2000 µg/mL did not cause toxic symptoms in tobacco [96] and in other plants [190]. This is probably due to the rapid breakdown of GABA.

## 3. Conclusions

Today, a prominent trend involves the integration of bioactive chemicals into eco-friendly, sustainable agriculture practices to stimulate plants’ inducible immune defense. These chemicals have a high efficacy in stimulating plant priming, which is a behavior characterized by numerous intricate pathways [59,61]. Priming compounds primarily focus on using naturally occurring substances that plants generate in response to stress. When applied externally, these protective chemicals can also be ecologically sustainable due to their demonstrated effectiveness, even at low levels. The use of NPPAs as priming agents is expanded because many studies indicate their distinct and broad protective benefits. Researchers recently discovered that AABA, once considered inactive in the early 2000s, can indirectly stimulate the onset of antioxidant defense responses. However, its effectiveness varies depending on the dosage, plant, and pathogen involved. According to our current point of view, it may be more effective against biotic stressors, but it certainly contains further unexploited research opportunities.

On the one hand, the cell-protective compound BABA induces the plant antioxidant system, and, on the other, it supports the production of a whole series of secondary metabolites during times of stress. Furthermore, it has a positive influence on photosynthesis processes and enhances cell membrane stability by facilitating the production of specific amino acids. It can not only have an impact in a specific area, but it can also trigger systemic acquired resistance (SAR) and sustain a stress memory. It efficiently triggers cellular detoxification activities, thereby positively impacting cellular physiological functions on multiple levels. Initially thought to be the most effective, BABA has proven its priming properties outstandingly in many fields of application, and we cannot ignore the fact that the third isomer, GABA, is currently receiving the most attention.

Similar to the study conducted by Wang et al. (2019) on BABA [108], Li et al. (2016) have demonstrated that GABA enhances AGC and plays a role in maintaining plant homeostasis by promoting a more robust function of the GABA shunt [150]. This increase in metabolism has a positive effect on the functioning of the citrate cycle, and, ultimately, on cellular respiration processes. In addition, it has been proven that GABA accumulated under certain stress conditions transports organic acids, such as succinate, to the citrate cycle [146,191]. It has also been proven that the GABA shunt performs important regulatory tasks not only in the case of abiotic but also in biotic stress effects by increasing antioxidant activity and regulating polyamine metabolism [192]. GABA is produced endogenously through the GABA shunt, and many stressors can induce its production. Several studies have confirmed that it has a significant priming effect when used as an exogenous compound [193]. In most plants, GABA appears to provide exceptional protection against various abiotic stresses, as it reduces oxidative damage by inducing antioxidant defense, increases the presence of osmolytes, and balances tissue turgor [194]. We can infer that all three NPAAs can effectively function as exogenous priming agents to counteract the detrimental impacts of both biotic and abiotic stressors. As eco-friendly materials, they can have a substantial effect on decreasing the ecological impact of foundational substances in the upcoming decades.

## Figures and Tables

**Table 1:** Demonstration of many plant species that have been examined to elucidate the function of ABAA in the tolerance and regulation of different types of stresses.

Plant Species	Biotic/Abiotic Stressor	References
Tobacco	tobacco mosaic virus	[95]
Tobacco	tobacco mosaic virus	[96]
Tomato	*Phytophthora infestans*	[100]
Tomato	fusarium wilt	[99]
Rapeseed	*Leptosphaeria maculans*	[101]
Grapes	*Leptosphaeria maculans*	[87]
Pear	*Penicillium expansum*	[102]
Pine	*Bursaphelenchus xylophilus*	[103]

**Table 2:** Demonstration of many plant species that have been examined to elucidate the function of BABA in the tolerance and regulation of different types of stresses.

Plant Species	Biotic/Abiotic Stressor	References
Sunflower	*Plasmopara helianthi*	[89]
Pepper	*Colletotrichum coccodes*	[90]
Grapes	*Plasmopara viticola*	[91]
Cauliflower	*Peronospora parasitica*	[92]
Arabidopsis	*Pseudomonas syringae* *Hyaloperonospora parasitica*	[107]
Lettuce	*Bremia lactucae*	[93]
Arabidopsis	*Pseudomonas syringae*	[104]
Arabidopsis	*Pseudomonas syringae* *Hyaloperonospora arabidopsidis*	[105]
Tomato	*Botrytis cinerea*	[106]
Grapes	*Botrytis cinerea*	[108]
Mango	*Colletotrichum gloeosporioides*	[109]
Peach	*Rhizopus stolonifer*	[110]
Arabidopsis	*Peronospora parasitica*	[111]
Pistachio	salt stress	[112]
Rapeseed	drought stress	[113]
Tobacco	chilling stress	[114]
Rapeseed	salt stress	[115]
Chickpea	salt stress	[116]
Chinese cabbage	heat stress	[117]
Linen	drought stress	[118]
Chickpea	drought stress	[119]
Pepper	drought stress	[120]
Arabidopsis	thermotolerance	[121]

**Table 3:** Demonstration of many plant species that have been examined to elucidate the function of GABA in the tolerance and regulation of different types of stresses.

Plant Species	Biotic/Abiotic Stressor/Other	References
Arabidopsis	cotton leafworm	[131]
Tomato	*Alternaria alternata*	[132]
Arabidopsis	*Botrytis cinerea*	[133]
Pepper	osmotic stress	[43]
Corn	salt stress	[134]
Tomato	salt stress	[135]
Creeping bentgrass	water stress	[136]
Black cumin	water deficit stress	[137]
White clover	salt stress	[138]
Barley	germination parameters	[139]
Mungbean	germination parameters	[140]
Pumpkin	storage parameters	[141]
Pumpkin	storage parameters	[142]
White clover	water stress	[143]
Chrysanthemum	heat stress	[144]
Creeping bentgrass	heat stress	[145]
Creeping bentgrass	water stress	[146]
Maize	water stress	[147]
Tomato	chilling stress	[148]
Creeping bentgrass	combined heat and drought stress	[149]
Creeping bentgrass	combined drought, heat, and salt stress	[145]
Creeping bentgrass	combined heat and drought stress	[150]
Sunflower	combined heat and drought stress	[151]
Lentil	heat stress	[152]
Bean	drought stress	[153]
Pepper	low light stress	[154]
Bean	osmotic, sulfur dioxide, salt stress	[155]
Mungbean	thermo protectant effect	[156]
Creeping bentgrass	heat stress	[110]
Mustard	chromium stress	[115]
Creeping bentgrass	aluminum stress	[157]
Apple	drought stress	[158]

## Data Availability

All data are available within the manuscript.
